# First report of *Giardia duodenalis* infection in bamboo rats

**DOI:** 10.1186/s13071-018-3111-2

**Published:** 2018-09-20

**Authors:** Xun Ma, Yi Wang, Hui-Jun Zhang, Hao-Xian Wu, Guang-Hui Zhao

**Affiliations:** College of Veterinary Medicine, Northwest A&F University, 22 Xinong Road, Yangling, 712100 People’s Republic of China

**Keywords:** *Giardia duodenalis*, Prevalence, Bamboo rat, Hunan Province

## Abstract

**Background:**

The zoonotic parasite, *Giardia duodenalis* (syns*. G. lamblia* and *G. intestinalis*), has been widely reported in humans and animals, including rodents. The bamboo rat, a rodent species belonged to the subfamily Rhizomyinae, is farmed in China because of its medicinal and edible values. However, no information of *G. duodenalis* infection was available in bamboo rats prior to the present study. Here, the prevalence and genetic diversity of *G. duodenalis* in bamboo rats from Hunan Province of China were investigated.

**Results:**

Of 480 faecal samples collected from six farms located in four cities (Wugang, Chenzhou, Huaihua and Jishou) of Hunan Province, 52 (10.8%) were positive for *G. duodenalis* infection by using a nested PCR approach targeting the beta giardin (*bg*) gene. Significant differences (*P* < 0.01) in prevalence were found among different age groups and geographical localities, and among different farms in Wugang city. Sequence analysis revealed existence of the zoonotic assemblage B and genetic diversity of *G. duodenalis* in these animals. Multilocus genotyping analysis also indicated broad genetic diversity of assemblage B isolates in these bamboo rats.

**Conclusions:**

This is the first report of the infection and genetic variations of *G. duodenalis* in bamboo rats. These findings will provide basic data for implementing effective strategies to control giardiasis in bamboo rats.

**Electronic supplementary material:**

The online version of this article (10.1186/s13071-018-3111-2) contains supplementary material, which is available to authorized users.

## Background

*Giardia duodenalis* (syns. *G. lamblia* and *G. intestinalis*) is a common zoonotic protozoan that inhabits the gastrointestinal tracts of animals and humans [1–3]. Giardiasis, caused by *G. duodenalis*, is an important diarrheal disease concerning both public and veterinary health worldwide [4, 5]. In recent decades, *G. duodenalis* infection has been widely reported in domestic (e.g. goats, pigs, cats, rabbits, dogs and calves) and wild animals (e.g. chipmunks, chinchillas, red foxes, lizards, aquatic birds, boars and rhesus macaque) in Asia, Europe, Australia, North America and Africa [6–19], and a wide distribution and high prevalence of up to 100% in some studies have been reported [20, 21]. Although the infection is commonly asymptomatic or self-limiting in immunocompetent hosts [22, 23], watery diarrhea and other significant clinical impacts due to *G. duodenalis* infection could be observed in young, undernourished, or immunocomprised individuals [24, 25]. Furthermore, dormant and long-lasting infectious cysts excreted from hosts can pollute food, water and the environment, causing food-borne and water-borne outbreaks for humans and animals [26, 27].

The bamboo rat (*Rhizomys sinensis*), a rodent species belonging to the subfamily Rhizomyinae, is widely distributed in southern Asia, i.e. southern China, India, Myanmar, northern Vietnam and Thailand [28, 29]. Because of the high protein content, low fat and cholesterol of bamboo rat meat [30], the medicinal and edible values of this animal are favored by people [30]. Since the 1990s, the artificial breeding of bamboo rats began and was rapidly developed [31]. In 2011, over 30 million bamboo rats were farmed in China [32], and the demand for meat of bamboo rats is increasing at an annual rate of 3% [31]. However, due to changes in habitats and food sources, various pathogens have been reported in bamboo rats [28, 29, 33, 34], and infections of these pathogens could seriously affect the health and economic significance of these animals [35, 36]. Furthermore, the transmission risk to people of some zoonotic pathogens (e.g. *Cryptosporidium* spp. [34], *Penicilliosis marneffei* [29], *Escherichia coli* [33], *Trichinella* spp.) in these animals should also not be neglected [37]. Although the prevalence of *G. duodenalis* in bamboo rats was, until now, unknown, the presence of this parasite in other rodents (e.g. rats, mice, rabbits, chipmunks, chinchillas, guinea pigs, beavers) has been reported in China, Romania, Laos, America, Germany, Austria, Denmark, Hungary, Finland, France, Italy, Luxembourg, the Netherlands, Norway, Poland, Portugal and Sweden, with a prevalence of 1.9–83.0% [9, 13, 14, 36, 38–42]. Among them, high prevalence was detected in chinchillas (55.7%) from Romania [14], chinchillas (61.4%) from European countries [42] and mice (83%) from Germany [40]. Of eight assemblages (A-H) reported in animals and humans [22, 40, 43], assemblage G has been detected in rats and mice [36], and some animal-specific (C-H) and zoonotic assemblages (A and B) were reported in other rodents, i.e. chinchilla, beaver, muskrat and rabbit [9, 26, 44, 45].

To reveal the infection status of *G. duodenalis* in farmed bamboo rats, the prevalence and assemblages of this parasite in bamboo rats from six farms in four cities of Hunan Province, China, were determined, and the genetic diversity of *G. duodenalis* was also investigated by using the multilocus genotyping technique based on three gene loci [46–48], namely beta giardin (*bg*), triosephosphate isomerase (*tpi*) and glutamate dehydrogenase (*gdh*).

## Methods

### Collection of faecal specimens

From August to October 2017, 480 fresh faecal samples (Additional file 1: Table S1), were collected from seemingly healthy bamboo rats in six farms located in four cities (Wugang, Chenzhou, Huaihua and Jishou) of Hunan Province (Additional file 2: Figure S1), south-central China. Bamboo rats within four age groups (< 6 months; 6–12 months; > 12–24 months; and > 24 months) were investigated in the present study. Because of similar housing conditions in all examined sampling sites in this study, a fraction of animals were included (Additional file 1: Table S1). Approximately a third of bamboo rats representing each age group were examined in Farms 1–4. Since animals belonging to limited age groups (Additional file 1: Table S1) were raised on Farm 5 and Farm 6, a small number of samples within the existed age groups were collected. A faecal sample from each animal was collected immediately after defecation using disposable plastic bags, marked with the age, date, site and number, then transferred into a 15 ml centrifuge tube with 2.5% potassium dichromate solution and stored at 4 °C.

### Extraction of genomic DNA from faecal samples

To remove potassium dichromate solution, all faecal specimens of bamboo rats were washed several times using distilled water under centrifugation at 13,000× *rpm* for 1 min. Then, the genomic DNA sample was extracted from approximately 300 mg of washed faeces using the commercial E.Z.N.A® Stool DNA Kit (Omega Bio-Tek Inc., Norcross, GA, USA) following the manufacturer’s protocol. The extracted DNA samples were stored at -20 °C for further analysis.

### Nested PCR analysis and sequencing

The prevalence of *G. duodenalis* in bamboo rats was determined by using a nested PCR targeting the *bg* gene using primers reported previously [7, 49–51]. The samples positive for the *bg* gene were further analyzed with nested PCRs of gene loci *tpi* and *gdh*, respectively [7]. A 25 μl PCR mixture was used for PCR amplification, containing 1 μl genomic DNA (for the primary PCR) or primary amplification product (for the secondary PCR) as the template, 2.5 μl 10× *Ex Taq* buffer (Mg^2+^ free), 2 mM MgCl_2_, 0.2 mM dNTP mixture, 0.625 U TaKaRa *Ex Taq* (TaKaRa Shuzo Co., Ltd, Dalian, China) and 0.4 μM of each primer. Negative and positive controls were included in each PCR reaction and negative samples were spiked with positive material to investigate whether sample material had been associated with inhibition. The secondary PCR amplicons were analyzed by using 1% agarose gels electrophoresis with ethidium bromide. The positive secondary amplicons were sent to Sangon Biotech Co. Ltd. (Shanghai, China) for direct sequencing using the secondary PCR primers.

### Sequence analysis

The nucleotide sequences obtained were aligned and edited by using the software Clustal X 1.83 [52]. Then, the corrected sequences were aligned with the reference sequences of *G. duodenalis* within GenBank by using Basic Local Alignment Search Tool (BLAST) of the National Center for Biotechnology Information (NCBI) to identify the assemblages and subtypes.

### Statistical analysis

The differences in prevalence among bamboo rats of different age groups and farms were calculated by using the Chi-square test within the software SPSS 19.0 for Windows (SPSS Inc., Chicago, IL, USA), and the difference was considered statistically significant when *P* < 0.05.

## Results and discussion

*Giardia duodenalis* infection has been reported in humans and a large number of animal hosts worldwide [8, 9, 15, 47]. The prevalence has been reported as 1.09–72.4% in farmed animals (e.g. rabbits, goats, cattle, pigs, dogs, cats, Tibetan sheep and calves), and 3.2–83.0% in wild rodents (e.g. *Apodemus* spp., *Microtus* spp., *Myodes* spp., house mice, brown rats, Asian house rats, beavers, roof rats and deer mice) [7, 9–12, 36, 38–41, 53–57]. In the present study, this parasite was detected in bamboo rats from Hunan Province of China, and 52 (10.8%) of 480 samples were positive for *G. duodenalis* infection based on the nested PCR amplification targeting the *bg* gene. The prevalence of *G. duodenalis* in bamboo rats in this study was well in the range of previous studies in farmed animals and wild rodents. However, significant differences in prevalence (*χ*^2^ = 59.031, *df =* 5, *P <* 0.01) were detected among six farms from four sampling cities, ranging from 0 to 38.2%. The highest prevalence was found on Farm 3 of Wugang city (38.2%), while no infections were detected on Farm 4 of Chenzhou city and Farm 6 of Jishou city (Table 1). Furthermore, the prevalence was also significantly different (*χ*^2^ = 28.876, *df* = 2, *P* < 0.01) among three farms in Wugang city, with the highest on Farm 3 and lowest on Farm 1 (Table 1). The differences in prevalence among these farms could be due to different feeding and management levels. For example, bamboo rats in Farm 4 from Chenzhou city were mainly fed with concentrate (e.g. corn, wheat) and kept in multilayer vertical cages, which reduced the risks of pathogenic infections. On the other hand, in farms of Wugang city, both fresh bamboo and concentrate were fed to the animals, all of which were reared in wooden fences. Furthermore, different sampling practices may also have contributed to the differences between farms. Samples varying in number were collected from different farms (Table 1), ranging from 15 (Farm 6) to 207 (Farm 1). Different age groups and sample numbers within the same age groups tested (Additional file 1: Table S1) are also possible explanations for differences in prevalence. Additionally, detection procedures and targets are also possible factors affecting the rates of *G. duodenalis* infection. Due to its multiple-copy nature [58], the small subunit ribosomal RNA (*SSU* rRNA) gene was commonly used as a good marker to detect *G. duodenalis* infection in animals and humans [22, 58, 59]. However, this gene locus cannot effectively identify assemblages and sub-assemblages because of low sequence variability [58]. In the present study, nested PCR targeting the *bg* gene was used to investigated *G. duodenalis* infection in bamboo rats. Theoretically, *bg* is unique to *Giardia* and has a high degree of sequence diversity for identifying sub-assemblages [49, 50, 58]. However, compared with the *SSU* rRNA gene locus, the copy number is lower. In addition, nested PCR may not be used to distinguish between infection (cysts being excreted following encystation) and *G. duodenalis* DNA being excreted due to dead cysts being ingested. Alternatively, although an under-estimation of prevalence may be associated with traditional microscopy [60], morphological methods could reflect the infection intensity and extent of environmental contamination [61–63]. A previous study also established a real-time method using the *bg* gene to simultaneously evaluate cyst viability and discern zoonotic *G. duodenalis* assemblages A and B [64]. Therefore, to fully reveal the infection status of *G. duodenalis* in bamboo rats, many methods should be incorporated in future studies.

**Table 1:** Prevalence and factors associated with *G. doudenalis* infection in bamboo rats in Hunan Province, China

Variable	Category	No. examined	No. positive (%)	Target locus (no. positive)		
				*bg*	*tpi*	*gdh*
Age	< 6 months	136	31 (22.8)	31	6	15
	6–12 months	128	8 (6.3)	8	4	6
	> 12–24 months	151	10 (6.6)	10	1	4
	> 24 months	65	3 (4.6)	3	1	2
	Total	480	52 (10.8)	52	12	27
Location	Wugang city (Farm 1)	207	19 (9.2)	19	8	15
	Wugang city (Farm 2)	66	9 (13.6)	9	2	3
	Wugang city (Farm 3)	55	21 (38.2)	21	2	9
	Chenzhou city (Farm 4)	111	0	0	0	0
	Huaihua city (Farm 5)	26	3 (11.5)	3	0	0
	Jishou city (Farm 6)	15	0	0	0	0
	Total	480	52 (10.8)	52	12	27

A significant difference (*χ*^2^ = 28.298, *df* = 3, *P* < 0.01) in prevalence was also observed in bamboo rats of the four age groups in the present study (Table 1). The infection seemingly decreased with age (Table 1), with the highest prevalence (22.8%) in animals under six months and the lowest (4.6%) in animals over 24 months, which is in concordance to previous studies that indicated that young individuals of humans and animals are prone for *Giardia* infection [25]. However, investigations in chinchillas [44] and rabbits [65–68] from China, and deer mouse from California central coast of the USA [38] were inconsistent with this common phenomenon. The difference could be caused by different detection technologies, sample numbers, feeding manners, environmental conditions and geographical separation.

Five *G. duodenalis* assemblages (A, B, D, E and G) have been detected in rodents [14, 36, 42, 45, 66, 69]. In the present study, all *G. duodenalis* positive samples from bamboo rats were identified as assemblage B. This finding would indicate the fact of simple *G. duodenalis* population in bamboo rats. This result may also be due to small sampling numbers from limited areas in this study. To address this point, a large number of epidemiological investigations should be implemented in future studies. However, assemblage B could be transmitted zoonotically [70–72] and has a broad host range, including livestock, companion animals, fish, and marine and wild mammals [12, 13, 18, 21, 26, 73, 74]. Furthermore, assemblage B was also detected in some water bodies [45]. Of the two assemblages (A and B) commonly found in humans, assemblage B was identified in over 50% of *Giardia*-infected children in some studies [22, 72, 75]. A significant association between diarrhoea and the infection of this assemblage was also reported in children from Havana, Cuba [76]. To address the zoonotic potential of *G. duodenalis* in bamboo rats in investigated areas of Hunan Province, the infection status of handlers/farmers in the farms should be determined in future studies.

Additionally, although previous studies have indicated that assemblage B isolates have high genetic polymorphism, double peaks and ambiguity SNPs to hinder the classification of sub-assemblages and subtypes [48], we did not find any ambiguous nucleotides in our study. Of 52 *G. duodenalis-*positive samples, 52, 12 and 27 were successfully amplified at loci *bg*, *tpi* and *gdh*, respectively, by using nested PCRs (Table 2). Six polymorphic sites were found at the *bg* gene locus and six distinct subtypes were identified (Table 2), including three known (named as Ba1, Ba2 and Ba3) and three novel (named as Ba4, Ba5 and Ba6) subtypes. The sequences of Ba1, Ba2 and Ba3 had a 100% identity to an isolate (KM977640) from chinchillas in China [44], an isolate (KY696836) from Hylobatidae in China [71], and an isolate (KM190805) from humans in Canada [45], respectively, while 99% identities of three novel subtypes Ba4, Ba5 and Ba6 were found with the reference sequence KM977640 [44]. Additionally, six subtypes (Bc1-Bc6) and four subtypes (named as Bb1-Bb4) were also identified in 27 *gdh*-positive samples and 12 *tpi*-positive samples (Table 2).

**Table 2:** Substitutions of intra-assemblage B in *bg*, *tpi* and *gdh* sequences

Subtype (*n*)	Nucleotide positions and substitutions						GenBank ID
*bg*	60	66	267	294	328	483	
Ref. sequence	G	T	C	C	G	C	KM977640
Ba1 (1)	G	T	C	C	G	C	MH598574
Ba2 (25)	G	C	C	C	G	C	MH598579
Ba3 (7)	A	T	C	T	G	C	MH598575
Ba4 (7)	G	C	T	C	G	C	MH598578
Ba5 (11)	G	C	C	C	G	T	MH598576
Ba6 (1)	G	C	C	C	A	C	MH598577
*tpi*	218	240	282	405	490		
Ref. sequence	C	G	C	A	G		HQ666898
Bb1 (6)	C	G	C	A	G		MH598587
Bb2 (4)	T	G	C	G	G		MH598589
Bb3 (1)	T	G	C	G	T		MH598588
Bb4 (1)	T	A	T	G	G		MH598586
*gdh*	192	282	369	396	477		
Ref. sequence	T	C	A	T	C		HM134212
Bc1 (2)	T	C	A	T	C		MH598585
Bc2 (8)	C	C	A	C	C		MH598582
Bc3 (4)	T	C	A	C	T		MH598583
Bc4 (3)	T	C	A	C	C		MH598581
Bc5 (8)	T	T	G	C	C		MH598580
Bc6 (2)	T	T	A	C	C		MH598584

## Conclusions

*Giardia duodenalis* occurs in bamboo rats from Hunan Province of China and the infection was significantly associated with age and farms. Although only one assemblage (B) was identified in bamboo rats in the present study, zoonotic potential and genetic diversity were found. To our knowledge, this is the first report on *G. duodenalis* infection in bamboo rats.

## Additional files

Additional file 1: **Table S1**. Sampling information in the present study. (DOCX 12 kb)

Additional file 2: **Figure S1**. Sampling sites in the present study. (TIF 2527 kb)

## Abbreviations

*bg*: beta giardin
*tpi*: triosephosphate isomerase
*gdh*: glutamate dehydrogenase
