# Supplementary material for: First report of Giardia duodenalis infection in bamboo rats
Source: Parasit Vectors. 2018 Sep 20;11:520. doi: 10.1186/s13071-018-3111-2 (PMC6149208; doi:10.1186/s13071-018-3111-2)
Supplement: Supplementary file 1 — Table S1. Sampling information in the present study. (DOCX 12 kb) [file 13071_2018_3111_MOESM1_ESM.docx]

| Farm | Sample number | | | | |
| --- | --- | --- | --- | --- | --- |
|  | > 6 months | 6 -12 months | > 12- 24 months | > 24 months | Total |
| 1 | 37 | 83 | 66 | 21 | 207 |
| 2 | 17 | 23 | 20 | 6 | 66 |
| 3 | 44 | 4 | 4 | 3 | 55 |
| 4 | 33 | 18 | 40 | 20 | 111 |
| 5 | 5 | 0 | 21 | 0 | 26 |
| 6 | 0 | 0 | 0 | 15 | 15 |

Table S1. Sampling information in the present study
