# Supplementary figures and images for: First report of Giardia duodenalis infection in bamboo rats
Source: Parasit Vectors. 2018 Sep 20;11:520. doi: 10.1186/s13071-018-3111-2 (PMC6149208; doi:10.1186/s13071-018-3111-2)

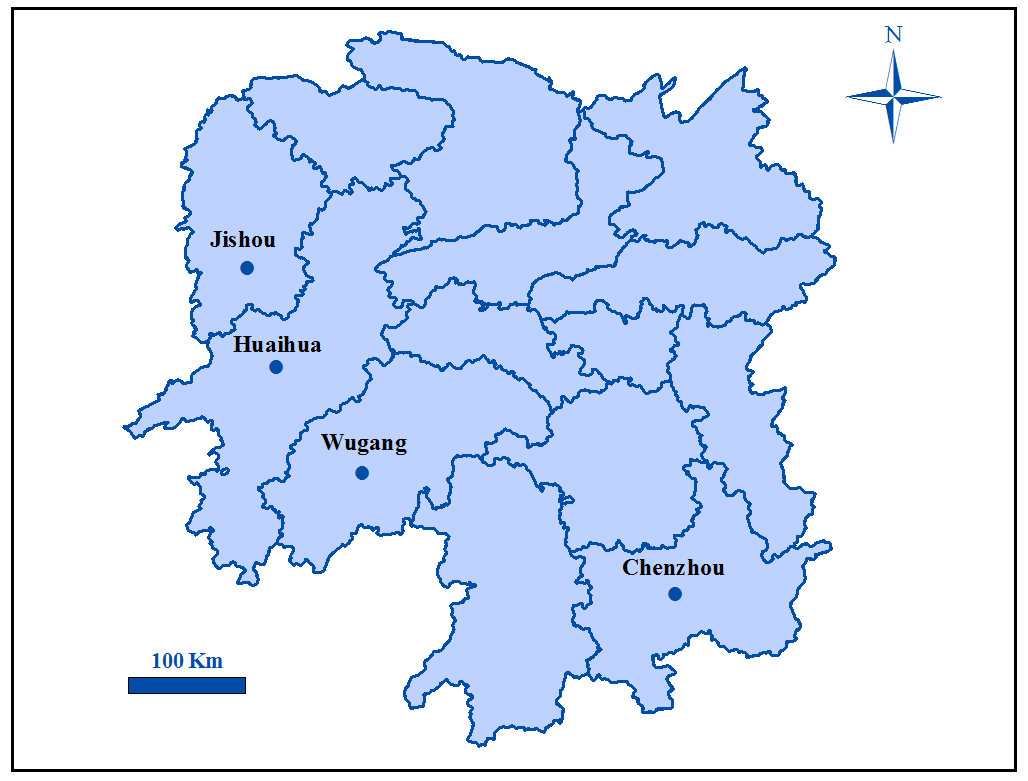

Supplement: Supplementary file 2 — Figure S1. Sampling sites in the present study. (TIF 2527 kb) [file 13071_2018_3111_MOESM2_ESM.tif]
